# The pathway to RCTs: how many roads are there? Examining the homogeneity of RCT justification

**DOI:** 10.1186/s13063-017-1804-z

**Published:** 2017-02-02

**Authors:** Jeffrey Tin Yu Chow, Kevin Lam, Abdul Naeem, Zarique Z. Akanda, Francie Fengqin Si, William Hodge

**Affiliations:** 1 Department of Epidemiology and Biostatistics, Schulich School of Medicine and Dentistry, The University of Western Ontario, London, Canada; 2 Schulich School of Medicine and Dentistry, The University of Western Ontario, London, Canada; 3 Faculty of Science, The University of Western Ontario, London, Canada; 4 Department of Ophthalmology, Ivey Eye Institute, St. Joseph’s Health Care London, London, Canada

**Keywords:** Randomized controlled trials, Clinical trials, Methodology, Justification, Evidence-based

## Abstract

**Background:**

Randomized controlled trials (RCTs) form the foundational background of modern medical practice. They are considered the highest quality of evidence, and their results help inform decisions concerning drug development and use, preventive therapies, and screening programs. However, the inputs that justify an RCT to be conducted have not been studied.

**Methods:**

We reviewed the MEDLINE and EMBASE databases across six specialties (Ophthalmology, Otorhinolaryngology (ENT), General Surgery, Psychiatry, Obstetrics-Gynecology (OB-GYN), and Internal Medicine) and randomly chose 25 RCTs from each specialty except for Otorhinolaryngology (20 studies) and Internal Medicine (28 studies). For each RCT, we recorded information relating to the justification for conducting RCTs such as average study size cited, number of studies cited, and types of studies cited. The justification varied widely both within and between specialties.

**Results:**

For Ophthalmology and OB-GYN, the average study sizes cited were around 1100 patients, whereas they were around 500 patients for Psychiatry and General Surgery. Between specialties, the average number of studies cited ranged from around 4.5 for ENT to around 10 for Ophthalmology, but the standard deviations were large, indicating that there was even more discrepancy within each specialty. When standardizing by the sample size of the RCT, some of the discrepancies between and within specialties can be explained, but not all. On average, Ophthalmology papers cited review articles the most (2.96 studies per RCT) compared to less than 1.5 studies per RCT for all other specialties.

**Conclusions:**

The justifications for RCTs vary widely both within and between specialties, and the justification for conducting RCTs is not standardized.

**Electronic supplementary material:**

The online version of this article (doi:10.1186/s13063-017-1804-z) contains supplementary material, which is available to authorized users.

## Background

Randomized controlled trials (RCTs) are often the cornerstone for deciding whether a specific medical intervention is used, yet there has not been much research concerning why each particular trial is conducted. As the name suggests, a randomized controlled trial is a trial where subjects are randomized to different groups, and after the intervention, the outcomes of the different groups are compared [1]. Randomizing the subjects ensures that, on average, other possible causes of the outcome are balanced between the groups and any significant differences between the outcomes of the different groups can be attributed to the intervention [2]. This helps to minimize bias and confounding in the trial.

Other than randomization, another essential component of an RCT is the control aspect. In a typical RCT, the outcomes of the intervention group are compared to a control group [3], where the intervention group receives the intervention and the control group receives no intervention or the current best practice intervention.

Since RCTs allow scientists and clinicians to make causal inferences between an intervention and its outcome, RCTs are one of the strongest forms of evidence for determining an intervention’s efficacy [3]. In evidence-based medicine, RCTs (and their systematic reviews) are positioned at the top of the hierarchy in terms of evidence used to guide medical decisions [4]. Not surprisingly, they are a staple of government regulatory bodies for decision making concerning drug applications to market.

Properly conducted RCTs can minimize bias and provide, as closely as possible, a definitive answer to the effectiveness of an intervention. With such an important type of evidence, naturally, there are rules for how to report the design and results of RCTs. While there are different guidelines available, the most commonly used and recommended is the Consolidated Standards of Reporting Trials (CONSORT) [5]. The CONSORT statement has recommendations for the reporting of every aspect of RCTs [5].

The conduct of an RCT, its reporting, and subsequent syntheses are well formalized, but the upstream process of any particular RCT is not formalized at all. There are typically two components to this upstream process. The “PICO” component (patient, intervention, comparison, outcome) is the creative process whereby the idea and the skeleton of the study are generated. The second upstream component we term “justification.” Justification is the body of previous information that allows for the conduct of an RCT given the need to justify issues of ethics, equipoise, and costs. The focus of this paper is to compare the upstream justification of RCTs both within and across different medical areas. Specifically, this paper will focus on what is recommended in the Introduction as the aim is to understand the justification for an RCT to be conducted.

Important information related to the scientific background and justification for the RCT should be included in the Introduction section of an RCT including any background or previous evidence for the benefits or harms of the interventions being studied in that particular trial [5]. One would also expect that the justification for conducting an RCT should be fairly uniform both within and across medical fields. In other words, one would expect that the justification for an RCT should be as uniform as its conduct, reporting, and subsequent syntheses.

Since RCTs are an important part of decision making for medical interventions, there should be a solid foundation and underlying evidence for an RCT to be conducted. With the increased regulations surrounding the conduct of RCTs, there have been increased costs associated with performing these trials [6]. Since RCTs are expensive to conduct, care should be taken to ensure that only relevant RCTs are performed. This means that there should be enough background evidence to justify the RCT, and one would hope for some sort of uniformity in RCT justification in general. Having an idea to answer a clinical question is not enough to justify conducting a RCT; there needs to be sufficient evidence in order to avoid conducting RCTs that waste money and unnecessarily place patients at risk.

RCTs are an essential part of decision making, so they should only be conducted when there is a solid base of evidence supporting their conduct. The present literature is very sparse at studying the reasons for why any particular RCT is being done. To formally study this question, this paper will quantify and summarize what types of evidence are cited in the Introduction as the reason for the RCT to be performed.

## Methods

### Study inclusion criteria

A literature search was conducted between January 2015 and July 2015 to identify randomized controlled trials (RCTs) published between January 2014 and July 2015 from the fields of Ophthalmology, Obstetrics and Gynecology (OB-GYN), Otorhinolaryngology (ENT), Internal Medicine, General Surgery, and Psychiatry.

Primary studies focusing on modeling or adverse events only were excluded, as were studies that included 20 or fewer participants. In addition, primary articles describing results from more than one trial were excluded.

### Search strategy

The search was conducted through OVID in the following databases: MEDLINE, EMBASE, and the Cochrane Central Register of Controlled Trials. The medical fields of concern were specified by exploding MeSH headings. A list of search terms used to search for articles in each field is provided in Additional file 1.

### Study selection

A random sample of 20–30 studies was chosen for data extraction in each medical field. Each study that was found through the search strategy was given a unique ID number. A complete list of unique ID numbers was then generated for each medical field in Microsoft Excel, and those studies were evaluated for the inclusion criteria. If a study did not meet the inclusion criteria, the next number in the list was evaluated for inclusion.

### Screening and data extraction

Two-level screening was performed by two reviewers — first with abstracts alone (level 1) and then with full-text articles (level 2). For the selected primary articles, basic study characteristics and patient demographics were collected: journal, print date, study objective, country of origin (as defined by primary author location), number of participants enrolled and the number completed, mean age of participants, gender (percentage of male participants), and race ethnicity (percentage of Caucasian participants).

Most importantly, the references in each study’s Introduction were examined. Only references used to justify conducting the RCT were included in data extraction. The references were categorized by the type of study. The sample size of the study referenced was recorded as well.

References were excluded if they discussed an earlier publication of the current trial or if they focused on topics peripheral to the study objective. References to descriptive information about the drug or condition being studied were also excluded.

The categories used for reference extraction were as follows: cited expert opinion, review articles, case reports or case series, case control studies, cohort studies, cross-sectional studies, and randomized controlled trials. For expert opinion and review articles, the sample size was not recorded.

To ensure consistent methodology, data extraction was performed by a primary reviewer and edited/confirmed by a secondary reviewer. Data extraction was performed by authors JC and KL, and disagreements were refereed by WH. Extracted data were recorded in an Excel spreadsheet.

## Results

Using the search strategy described above, the following numbers of RCTs for each specialty were found: Ophthalmology (156), OB-GYN (99), Psychiatry (81), General Surgery (113), ENT (27), and Internal Medicine (1016). Twenty-five RCTs were randomly selected from each specialty using the RANDOM function in Microsoft Excel. However, only 20 RCTs from ENT were included in the analysis due to the lack of RCTs meeting inclusion/exclusion criteria, and 28 RCTs from Internal Medicine were included due to the need to select an equal number of studies from each of the seven subspecialties for Internal Medicine (Cardiology, Endocrinology, Gastroenterology, Hematology, Nephrology, Pulmonology, and Rheumatology).

Table 1 outlines the patient demographics from the RCTs used (not the cited studies). The lowest average age was found in Psychiatry studies (39.1 years), with Internal Medicine, Ophthalmology, and General Surgery having higher average age patients, typically in their mid-50s. ENT had 70% males in their RCT studies; of course, OB-GYN had 100% females. The rest of the specialties had close to a 50/50 split between genders. Approximately three quarters of the studies across all specialties had Caucasians as the predominant race for participants.

**Table 1:** Demographic characteristics stratified by specialty

Specialty		Mean age (years)	Gender (% Male)	Race (% White)
Ophthalmology	AVERAGE	58.3	44.7	78.2
	Std. Dev.	13.3	16.0	14.9
	Maximum	80.0	100	96.8
	Minimum	36.7	24.1	53.1
Obstetrics and Gynecology	AVERAGE	45.7	0	75.0
	Std. Dev.	16.0	0	28.5
	Maximum	63.7	0	97.1
	Minimum	23.0	0	0
Psychiatry	AVERAGE	39.1	50.7	75.6
	Std. Dev.	17.7	22.4	14.5
	Maximum	75.5	100	97.0
	Minimum	8.8	0	50.8
General Surgery	AVERAGE	57.0	51.7	81.5
	Std. Dev.	11.5	24.4	15.8
	Maximum	78.1	80.5	96.4
	Minimum	38.0	0	49.2
Otorhinolaryngology	AVERAGE	43.9	69.6	73.0
	Std. Dev.	16.7	18.7	29.4
	Maximum	64.8	96.8	97.9
	Minimum	7.2	25.0	0
Internal Medicine	AVERAGE	53.2	53.9	76.4
	Std. Dev.	17.6	20.2	15.8
	Maximum	71.5	78.3	98.0
	Minimum	6.1	0.1	47.5

*Std. Dev.* standard deviation

**Fig. 1:**
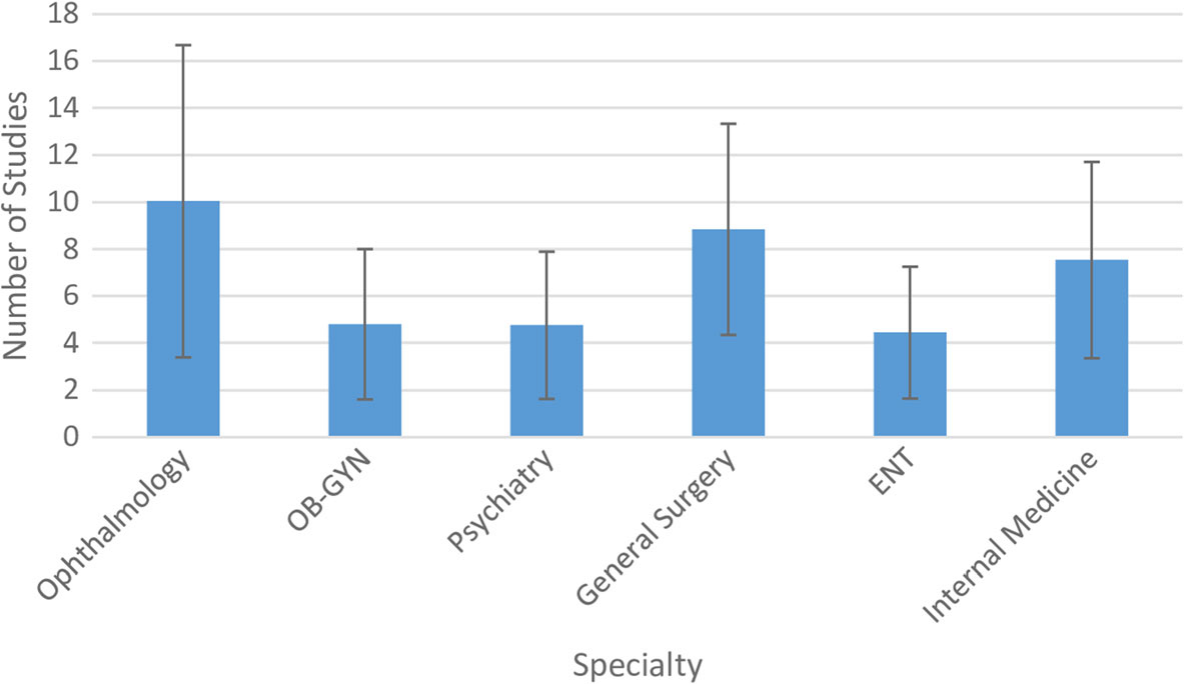
Average number of studies cited to justify RCTs stratified by specialty

Figure 1 shows the average total number of studies cited per specialty to justify the present published RCT. ENT had the lowest number of cited studies at 4.5 and Ophthalmology the highest at 10.0. While this is more than a twofold range, the discrepancy within specialties was even greater. For example, in Ophthalmology, as few as 2 articles were cited to justify some RCTs and as many as 27 to justify others. Clearly, both within and across medical areas, the number of cited studies used to justify an RCT varies widely.

Table 2 summarizes the average number of studies cited per RCT in each specialty and also breaks this number down by study type. Cohort studies would typically be considered the highest quality evidence after RCTs. There was again a large range difference for cohort studies cited between medical fields and an even larger range within medical fields. Between medical fields, Obstetrics/Gynecology RCTs cite on average 0.2 cohort studies per RCT done and Internal Medicine RCTs cite 1.3 cohort studies per RCT done, which is more than a sixfold difference. Within medical specialties, the range is large and the standard deviation is always larger than the mean, indicating a very large discrepancy in the type of study cited within medical areas.

**Table 2:** Total number of studies cited to justify RCTs stratified by specialty and study type

Specialty		Total papers	Case series	Case controls	Cohort studies	Cross sectional	RCTs	Expert opinion	Review articles
Ophthalmology	AVERAGE	10	1.16	0.24	1.1	0.68	3.52	0.24	2.96
	Std. Dev.	6.6	2	0.83	1.5	1.35	3.83	0.52	2.42
	Maximum	27	9	4	5	5	18	2	7
	Minimum	2	0	0	0	0	0	0	0
OB-GYN	AVERAGE	4.8	1.7	0	0.2	0.04	1.96	0.12	0.68
	Std. Dev.	3.2	2.4	0	0.5	0.2	2.13	0.33	1.07
	Maximum	12	8	0	2	1	7	1	4
	Minimum	0	0	0	0	0	0	0	0
Psychiatry	AVERAGE	4.8	0.24	0.04	0.4	0.36	2.52	0.04	1.16
	Std. Dev.	3.1	0.66	0.2	1.4	0.99	2.02	0.2	1.46
	Maximum	12	3	1	7	4	7	1	5
	Minimum	0	0	0	0	0	0	0	0
General Surgery	AVERAGE	8.8	2	0.16	0.88	0.16	3.64	0.6	1.40
	Std. Dev.	4.5	2	0.47	0.88	0.47	3.17	1.08	1.41
	Maximum	21	7	2	3	2	12	4	6
	Minimum	4	0	0	0	0	0	0	0
ENT	AVERAGE	4.5	0.95	0.05	0.25	0.15	1.85	0.05	1.05
	Std. Dev.	2.8	1.1	0.22	0.91	0.49	2.25	0.22	1.36
	Maximum	11	4	1	4	2	9	1	5
	Minimum	0	0	0	0	0	0	0	0
Internal Medicine	AVERAGE	7.5	0.93	0.21	1.29	0.39	3.18	0.25	1.11
	Std. Dev.	4.2	1.6	0.69	2.42	0.99	3.24	0.65	1.07
	Maximum	19	7	3	10	4	10	3	3
	Minimum	2	0	0	0	0	0	0	0

*RCT* randomized controlled trial, *Std. Dev.* standard deviation, *OB-GYN* Obstetrics and Gynecology, *ENT* Otorhinolaryngology

**Fig. 2:**
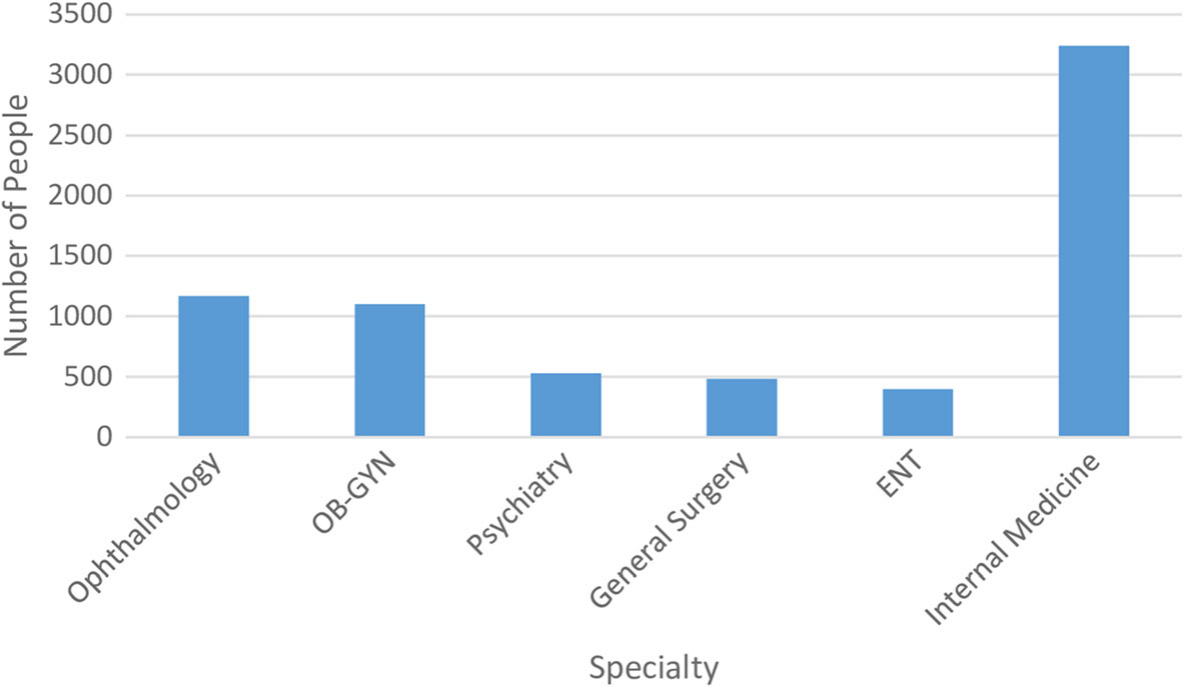
Average size of study cited stratified by specialty

The average size of studies cited also varies widely. It should be noted that in all medical areas, it is quite common to cite population-based studies as reasons to justify RCTs; hence, the average study sizes tend to be large across all fields. However, the differences are again very marked. Figure 2 summarizes this data. The average ENT study cited involved 399 participants, while in Internal Medicine the size was 3238 patients, an eightfold difference. And within Internal Medicine, the range varies from 34 patients to 62,134 participants, a more than 1800-fold difference.

One might hypothesize that part of the reason for the discrepancy in study size cited and number of cited studies to justify RCTs both within and between medical fields may have to do with trends for RCT sizes in each field. In other words, one might hypothesize that Internal Medicine RCTs are typically larger and performed more frequently than Ophthalmology or ENT studies, and this may explain part of the differences. In order to study this, we standardized both cited study number and cited study size by the RCT “*N*” or number enrolled. These data are provided in Table 3. As can be seen in columns 5 and 6, this standardization does explain *some* of the between-specialty differences. For example, standardized study size cited/*N* enrolled (column 5) shows converging values for Ophthalmology, OB-GYN, Psychiatry, and General Surgery. In the final column, total numbers of studies cited are standardized by *N* enrolled, and converging values are found for ENT, Internal Medicine, OB-GYN, and Ophthalmology. While standardizing in this fashion explains some between-specialty differences, there is still much discrepancy remaining, and not all of the within-specialty discrepancies can be accounted for by standardization.

**Table 3:** Average cited study size and average number of studies cited standardized by RCT size

Medical area	*N* enrolled	Average cited study size	Average number of studies cited	Cited study size/*N* enrolled	Number of studies cited/*N* enrolled
Ophthalmology	1060	1167	10.0	1.10	0.009
OB-GYN	618	1099	4.8	1.78	0.008
Psychiatry	347	530	4.8	1.53	0.014
ENT	2712	399	4.5	0.15	0.002
General Surgery	362	482	8.8	1.33	0.024
Internal Medicine	1192	3238	7.5	2.72	0.006

*N* number, *RCT* randomized controlled trial, *OB-GYN* Obstetrics and Gynecology, *ENT* Otorhinolaryngology

## Discussion

Randomized clinical trials (RCTs) provide results that form the backbone of modern medical practice. Because their results are so important, it is not surprising that their conduct and reporting are highly standardized. The CONSORT Statement [5] is an internationally recognized guide to the conduct and reporting of RCTs. The statement includes recommendations for all aspects of RCT conduct and reporting, such as inclusion criteria, randomization, stopping rules, and analysis.

However, the justification for why we conduct RCTs is not evidence-based. There is no Prestudy CONSORT or equivalent guide that recommends the level of evidence needed to conduct an RCT in the first place. Some RCTs are conducted after a rigorous analysis of many evidence-based observational studies and small preliminary clinical trials. Others are conducted based on case series and expert opinion.

Our study has quantified the wide discrepancy in evidence that is used to justify the conduct of RCTs. We randomly compared this evidence across six medical areas and within these areas as well. We randomly picked approximately 25 papers per specialty and studied the justification explanation in the Introduction of each RCT. We tabulated the number of studies that justified each RCT, the size of the studies that were used to justify the RCT, and the type of study as well (including cited expert opinion).

Although the discrepancies found to justify RCTs were large across all outcomes studied, Table 2 summarizes the large discrepancy found when looking at type of study cited to justify RCTs. Across specialties there is a large range of study types used to justify RCTs. The only area where there is reasonable convergence is previous RCTs cited (range 1.85–3.64). For all other study types there is a large range of study numbers used to justify an RCT. The mix of studies used to justify RCTs also varies widely. Finally, the large standard deviations in Table 2 (often the standard deviations are larger than the mean) show that discrepancies justifying RCTs are even larger within specialties than they are across specialties.

Since systematic reviews and meta-analyses are used to synthesize existing evidence available for a particular treatment or intervention and identify potential gaps where future research is needed, naturally, they are useful and necessary for justifying the conduct of an RCT [7]. However, many researchers often fail to consider the results of relevant systematic reviews when designing their RCTs [8]. Our study found that RCTs did not consistently have systematic reviews or meta-analyses cited as a justification for the study to be conducted since there were large discrepancies both between and within specialties. A previous study looking at trials published in leading medical journals in 2007 found that, in the Introduction section of each trial, 56% of trials referred to at least one previous individual trial, 22% referred to at least one meta-analysis, and 7% referred to at least one systematic review [9]. In contrast, Cooper at al. found that 33% of trials had used a systematic review to influence their study design, but this difference may have been due to Cooper et al. only including trials that had a relevant Cochrane review available in the literature [10].

Potential limitations of our study include a lack of classification of RCTs and the number of RCTs included. In our study, we did not distinguish between RCTs that were clinical trials from different phases. Although all studies we included were randomized, different clinical trial phases are intended to answer different research questions, so their justification to be conducted may also be different. For example, some phase III trials are intended to determine the efficacy of a new treatment compared to the standard treatment, while some phase II trials are intended to determine the optimal dose while balancing toxicity and efficacy [11]. At earlier phases, different types and levels of evidence may be available to justify the conduct of an RCT [11], but this was not taken into account in our study. However, the differences and progression between different clinical trial phases are not always clearly defined or consistent [12], so treating RCTs as one large group may be the best possible way to study the issue of RCT justification.

The number of studies included is also a potential limitation, as only 148 RCTs were included in the analysis. There are many other RCTs available in the literature, so the sample size may not have been large enough, and the results of this study should be considered exploratory. This may also be another reason why the standard deviations were so large in the analysis. However, randomization was used to select studies in order to prevent potential bias in selecting RCTs.

## Conclusions

Because the use of RCTs is so widespread in modern medicine, we feel that the reasons justifying RCTs should also be more convergent. Since the justification for conducting RCTs varies widely both within and across specialties, there is a need for a set of guidelines to be developed in order to help investigators decide when there is sufficient evidence to conduct an RCT. This is an area that researchers, clinicians, and policy makers should work toward together in the not too distant future.

## Additional file

Additional file 1: Search terms used in search strategy. A description of the search terms used in the search strategy for each specialty. (DOCX 14 kb)

## Abbreviations

CONSORT: Consolidated Standards of Reporting Trials
ENT: Otorhinolaryngology
OB-GYN: Obstetrics-Gynecology
RCT: Randomized controlled trial
