# Supplementary material for: The pathway to RCTs: how many roads are there? Examining the homogeneity of RCT justification
Source: Trials. 2017 Feb 2;18:51. doi: 10.1186/s13063-017-1804-z (PMC5288880; doi:10.1186/s13063-017-1804-z)
Supplement: Additional file 1: — Search terms used in search strategy. A description of the search terms used in the search strategy for each specialty. (DOCX 14 kb) [file 13063_2017_1804_MOESM1_ESM.docx]

**Additional file 1** Search terms used in search strategy

| **Medical field** | **MEDLINE**  **(MeSH)** | **EMBASE**  **(EMTREE)** |
| --- | --- | --- |
| *Ophthalmology* | exp Eye Diseases/ | exp Eye Disease/ |
|  | exp Ophthalmologic Surgical Procedures/ | exp Eye Surgery/ |
|  |  | plus |
|  |  | Embase Section Heading: Ophthalmology |
| *Otolaryngology* | exp otorhinolaryngologic diseases/ | exp "ear nose throat surgery"/ |
|  | otorhinolaryngologic surgical procedures/ | exp "ear nose throat disease"/ |
|  |  | plus |
|  |  | Embase Section Heading: otorhinolaryngology |
| *Internal Medicine* | exp Rheumatic Diseases/ | exp rheumatic disease/ |
|  | exp Digestive System Diseases/ | exp digestive system disease/ |
|  | exp Respiratory Tract Diseases/ | exp respiratory tract disease/ |
|  | exp Kidney Diseases/ | exp kidney disease/ |
|  | exp Heart Diseases/ | exp heart disease/ |
|  | exp Hemic and Lymphatic Diseases/ | exp hematologic disease/ |
|  | exp Endocrine System Diseases/ | exp endocrine disease/ |
|  | NOT | plus |
|  | (subject headings used for other specialities listed here) | Embase Section Heading: Internal Medicine |
|  |  | NOT |
|  |  | (subject headings used for other specialities listed here) |
| *General Surgery* | exp bariatric surgery/ |  |
|  | exp digestive system surgical procedures/ | exp abdominal surgery/  (incl. GI and bariatric surgeries) |
|  | exp endocrine surgical procedures/ | exp endocrine surgery/ |
|  | exp dermatologic surgical procedures/ | exp skin surgery/ |
|  | kidney transplantation/ | exp kidney transplantation/ |
|  | liver transplantation/ | exp liver transplantation/ |
|  | pancreas transplantation/ | exp pancreas transplantation/ |
|  |  | exp intestine transplantation/ |
|  |  | parathyroid transplantation/ |
|  |  | spleen transplantation/ |
|  |  | exp thymus transplantation/ |
|  |  | exp general surgery/ |
|  |  | exp breast surgery/ |
|  |  | exp pelvis surgery/ |
| *Psychiatry* | exp Mental Disorders/ | exp mental disease/ |
|  |  | plus |
|  |  | Embase Section heading: psychiatry |
| *OB/GYN* | exp "female urogenital diseases and pregnancy complications"/ | exp "genital system disease"/ |
|  | exp Obstetric Surgical Procedures/ | exp "pregnancy disorder"/ |
|  | exp Gynecologic Surgical Procedures/ | exp "obstetric operation"/ |
|  |  | exp "gynecologic surgery"/ |
|  |  | plus |
|  |  | Embase Section Heading: "obstetrics and gynecology" |
